# Survival Impact of Textbook Oncological Outcomes and SDHs for Patients with Operable Esophageal Cancer

**DOI:** 10.3390/cancers18081253

**Published:** 2026-04-15

**Authors:** Ahmed Alnajar, Nestor Villamizar, Mehmet Akcin, Dao M. Nguyen, Diego Avella-Patino

**Affiliations:** 1 DeWitt Daughtry Department of Surgery, University of Miami Health System, Miami, FL 33136, USAdnguyen4@med.miami.edu (D.M.N.); 2 Division of Thoracic Surgery, DeWitt Daughtry Department of Surgery, University of Miami Health System, Miami, FL 33136, USA; nvillamizar@med.miami.edu

**Keywords:** social determinants of health, textbook oncological outcomes, esophageal cancer, surgical quality, survival disparities

## Abstract

**Background**: Disparities in esophageal cancer (EC) outcomes persist, partially due to inequitable access to high-quality surgical care, particularly for socioeconomically disadvantaged populations. This study evaluates the impact of social determinants of health (SDHs) on achieving textbook oncological outcomes (TOOs) and survival in operable EC patients. **Methods**: Using the National Cancer Database (2010–2021), we analyzed 26,367 stage I–IV A esophageal adeno- and squamous cell carcinoma patients undergoing esophagectomy after neoadjuvant chemoradiation. An SDH score (0–4) was derived from income, education, rurality, and hospital type. TOOs were defined as R0 resection, ≥20 lymph nodes examined, no 30-day mortality, and no prolonged hospitalization. Multivariable logistic and Cox regression models assessed predictors of TOOs and survival. **Results**: Overall, 19% achieved TOOs. Median survival time was 6.4 years for TOO+ versus 3.2 years for TOO−. Patients with favorable SDH had longer survival than those with unfavorable SDHs (median 4.0 vs. 3.5 years), but this disparity was largely confined to TOO− patients. Across stages II–III, low income and treatment at community hospitals were consistently associated with higher mortality among TOO− patients (HRs ~1.08–1.21), whereas SDH factors were largely attenuated among TOO+ patients. Stage-stratified analyses demonstrated that SDH effects were minimal in stage I and IV disease but pronounced in intermediate stages, especially in TOO− patients. **Conclusions**: TOOs are a powerful modifier of survival disparities in esophageal cancer. While SDH strongly influences outcomes among patients with suboptimal care pathways, achieving high-quality, textbook oncologic care attenuates these effects. Efforts to improve TOO achievement and expand access to high-quality centers may reduce socioeconomic inequities in survival.

## Simple Summary

Esophageal cancer remains associated with poor survival despite advances in surgery and cancer therapy. Outcomes vary widely, and patients from socially disadvantaged backgrounds often experience worse results. This study evaluated whether differences in income, education, and access to specialized hospitals influence survival after surgery for esophageal cancer, as well as whether receiving optimal, high-quality care can reduce these disparities. We found that patients who completed an ideal course of surgical and postoperative care had substantially better survival, regardless of social background. However, patients who did not receive optimal care were under the most significant effect of social disadvantage. These findings suggest that improving access to high-quality surgical treatment and supporting vulnerable patients throughout their care journey may help reduce survival disparities and improve outcomes in esophageal cancer.

## 1. Introduction

Esophageal cancer (EC) is a global health crisis, ranking sixth in cancer-related mortality worldwide, with over 600,000 new cases diagnosed annually [1,2]. Despite advancements in multimodal therapy, including neoadjuvant chemoradiation and minimally invasive surgical techniques, 5-year survival remains dismal at 40% for locally advanced disease [3]. Differences in survival outcomes persist in part due to inequities in access to high-quality surgical and medical care, disproportionately affecting socioeconomically disadvantaged populations [2].

Textbook oncological outcomes (TOOs) have been utilized as a surgical quality metric, reflecting the successful completion of an ideal perioperative and oncologic care pathway. TOOs incorporate key domains, such R0 resection, adequate lymphadenectomy, and absence of major complications [4,5]. Prior studies have demonstrated that TOOs correlate with a significant improvement in EC survival [6,7]. However, only a few patients achieve TOOs in real-world practice, and substantial disparities in TOO attainment persist across patient populations and treatment settings. For example, Medicaid recipients and uninsured patients achieve TOOs only half as often as privately insured patients [7]. Thus, social determinants of health (SDHs), including income, education, rurality, and insurance status, have been shown to be critical yet understudied drivers of postoperative outcomes. For instance, low-income patients are less likely to receive definitive surgical or multimodality treatment and experience delays to surgery that are often 3–4 months longer than those affluent counterparts, contributing to their higher rates of complications and mortality [3,8]. Similarly, rural residents face systemic barriers based on lack of access to high-quality surgical care [9,10]. Furthermore, the high-volume centers treating EC are more frequently located in urban areas, which introduces new challenges to access [9]; thus EC surgery at high-volume centers has been shown to reduce mortality and complication rates, yet access to such centers remains uneven, potentially exacerbating existing disparities [9,11].

While prior studies have independently linked SDHs, surgical volume, and TOOs to survival, the intersection between SDHs and TOOs across disease stages remains poorly understood. Specifically, it is unknown whether achieving high-quality, textbook oncologic care can mitigate the adverse survival effects associated with socioeconomic disadvantage or whether SDHs continue to exert a detrimental influence even among patients receiving optimal surgical care.

Accordingly, this study examines the interplay between SDHs, achievement of TOOs, and overall survival among patients with EC treated at National Cancer Database-participating hospitals. By integrating stage-stratified and TOO-stratified analyses, we aim to clarify how both social disadvantage and care quality influence long-term survival. With this analysis, we aim to identify actionable targets for reducing inequities in EC outcomes.

## 2. Materials and Methods

### 2.1. Data Source

The data used in the study are derived from the deidentified esophageal participant user data file 2022 of the National Cancer Database (NCDB). The NCDB is a hospital-based cancer registry that is a joint program of the American College of Surgeons Commission on Cancer and the American Cancer Society. The University of Miami Miller School of Medicine’s institutional review boards deemed this study exempt from review because the data were deidentified.

### 2.2. Study Population

Inclusion criteria included adult (≥18 years) patients from 2010 to 2021 with tumors with adenocarcinoma or squamous cell carcinoma, clinical stage I–IVA, who underwent esophagectomy (Surveillance, Epidemiology, and End Results codes: 30, 40, 50:55) and did not have palliative surgery. Exclusions included metastatic disease, no induction provided when patients were in stage II–IVa, as indicated by the guidelines, and incomplete follow up records, as presented later in the consort diagram. We included stage IVa patients who underwent esophagectomy for the completeness of the analysis [12]. However, this is a highly selective group of patients which may not reflect the overall survival of stage IV EC patients. However, they were included to reflect real-world surgical cohorts within the registry, as it captures a large sample size and provides answers that otherwise cannot be answered, rather than including them to represent the entire stage IV population.

### 2.3. Study Outcomes and Covariates

The primary outcome was overall survival, which was defined as the time from the date of diagnosis to the date of death from any cause. The main variables were the achievement of TOOs and SDHs. SDHs were constructed from residential zip-code data, while TOOs were constructed from the treating facility data. Thus, residential SDHs do not necessarily reflect the resource level of the treating institution.

TOO was defined as a composite measure, including:

- No 30-day mortality;
- No readmission within 30 days;
- Negative surgical margins;
- At least 20 lymph nodes examined;
- No prolonged hospital stay (should be ≤14 days).

This definition is based on previously published literature describing textbook outcomes in surgical oncology [4,7,13,14]. Patients who met all criteria were classified as TOO+, while those who failed any component were classified as TOO−.

An SDH score (0–4) was a metric constructed using the item-response theory-based method, defined a priori based on prior thoracic oncology literature and our previously published work. It was derived from four variables:

1. **Income**: lowest quartile (≤$38,000/year) = 1 point.
2. **Education**: <high school diploma in patient’s residential area = 1 point.
3. **Rurality**: rural area as primary residence = 1 point.
4. **Limited access to specialized care**: community hospital < 250 miles = 1 point.

Based on the total score, patients were stratified as presenting unfavorable SDHs (score ≥ 2) or favorable SDHs (score < 2). This composite score was defined and validated before in thoracic [15,16] and endocrine [17,18] cancers. Each SDH factor was examined individually and as part of a composite score. SDH burden was summarized for each patient and stratified across clinical stages and TOO status.

Other variables of interest included demographics (age, sex, race, and/or ethnicity, grouped into Hispanic and non-Hispanic [White, Black, Asian, or other]), Charlson–Deyo comorbidity index (CDCI), facility type (academic, integrated, community programs regardless of the distance from the patients’ location [comprehensive or otherwise]), and facility esophageal cancer case volume (categorized into low and high by the median yearly case volume).

### 2.4. Statistical Analysis

Baseline characteristics were summarized using descriptive statistics. Frequencies of SDH variables were compared across clinical stages and between TOO+ and TOO− patients using chi-square tests. Missing values were handled using mode and median imputations.

We then performed survival analysis using Kaplan–Meier curves to visualize differences in overall survival by SDH status within each clinical stage, stratified by TOO status. These curves were generated separately for TOO+ and TOO− groups. Comparisons were made using the log-rank test.

To examine the independent association between each SDH factor and overall survival, we constructed Cox proportional hazards models for each SDH variable stratified by clinical stage and TOO status. These models were adjusted for clinical data that affect disease severity and the likelihood of achieving TOOs, such as age at diagnosis, histological type, cancer location, Charlson–Deyo comorbidity score, and stage, in addition to other relevant variables, such as insurance type (private, Medicaid, Medicare/other government), and facility volume. These models excluded used the imputed dataset. Since stage IVa patients represent a highly selected subgroup, we perfumed additional sensitivity analyses excluding them. For each SDH factor, hazard ratios (HRs) and 95% confidence intervals (CIs) were extracted and plotted by stage and TOO status. Forest plots were created for each SDH variable (poor income, low education, rural area, community hospital access) and presented as grouped figures.

Finally, a multivariable Cox model was run stratified by TOO status, including all SDH variables simultaneously to assess their relative impact on survival. This final model was also adjusted for the covariates mentioned above, and the HRs were visually plotted in figures.

All statistical tests were two-sided, and a *p*-value < 0.05 was considered statistically significant. Analyses were conducted in R version 4.4.3 using the ‘survival’, ‘survminer’, ‘gtsummary’, and ‘ggplot2’ packages.

## 3. Results

### 3.1. Study Cohort Overview

**Figure 1:**
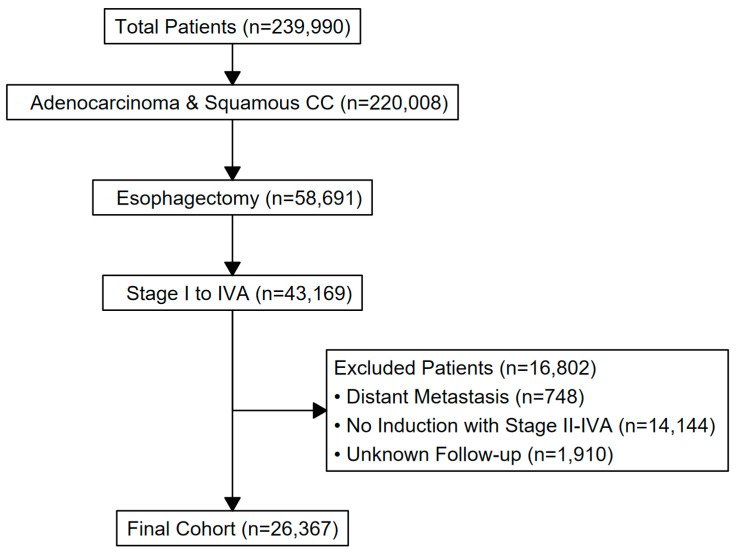
Consort diagram for inclusion and exclusion criteria.

A total of 26,367 patients fulfilled the criteria of this study (Figure 1). The cohort’s mean age was 63 years (±9), and 84% was male. The cohort was 91% non-Hispanic White; Black, Hispanic, and Asian patients accounted for 4.0%, 2.9%, and 1.5% of the cohort, respectively. Insurance status included private (45%), Medicare/other government (47%), and Medicaid/uninsured (7.6%).

TOO+ patients made up 19% of the cohort. More specifically, 20% of adenocarcinoma cases were TOO+, whereas of all squamous cases, 15% were TOO+ (*p* < 0.001). Overall stage distribution was 6.3% I, 34% II, 55% III, and 4.4% Iva, with TOO+ cases rising from 16% (stage I) to 26% (stage IVa) (*p* < 0.001). Procedures were most often performed at academic centers (50%), then community (32%) and integrated (18%) centers. The patients who achieved TOO+ status were 22% at academic vs. 16% at community and 16% at integrated programs (*p* < 0.001). High-volume hospitals treated 64% of all patients and had 21% TOO+ cases, while at low-volume centers 15% were TOO+ (*p* < 0.001). These results are presented in Table 1.

Across stages in Table 2, the percentages of TOO achievement lay the groundwork for the subsequent stage-stratified and multivariable analyses to its stage. The poor income and community hospital care factors were consistently lower among TOO+ than TOO− patients (e.g., stage I: 64% vs. 71% poor income; 26% vs. 34% community), low education was common in both groups (~75–81% vs. ~80–82%), and rural residence remained rare (<4%) with minimal differences.

### 3.2. Primary Outcomes

**Figure 2:**
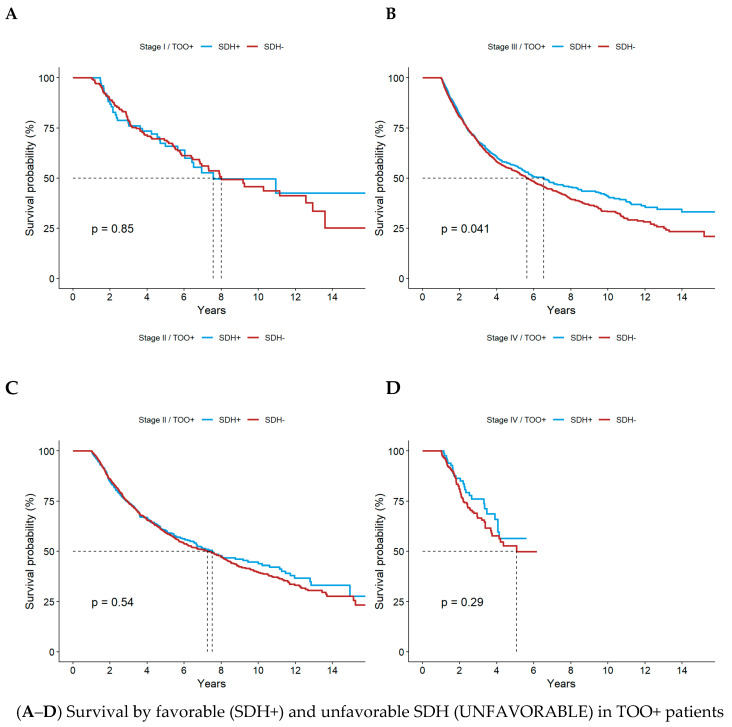

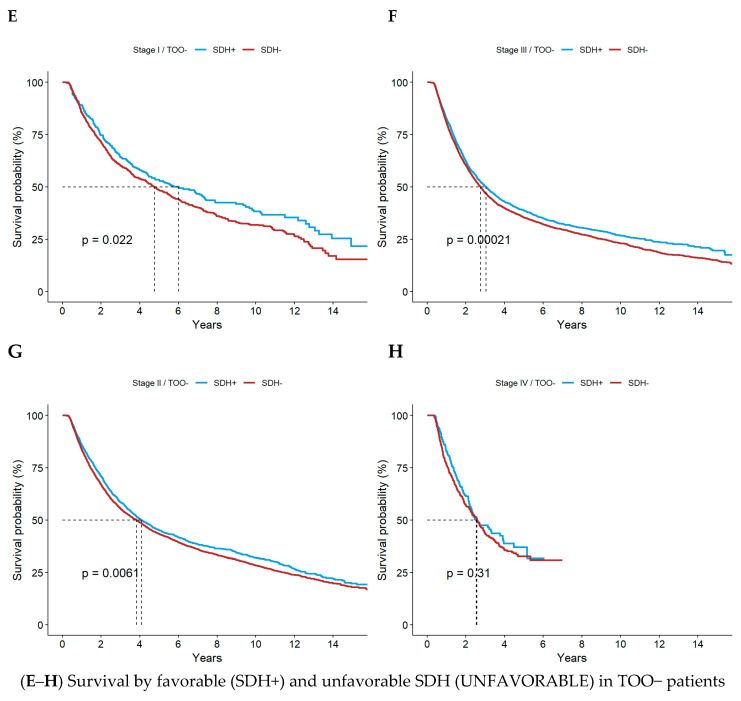
KM Curves by SDH status, stratified by TOO and stage (8 panels). Footnote: Panels (**A**–**D**) depict overall survival among patients who achieved textbook oncological outcomes (TOO+) stratified by SDH status (favorable [+] vs. unfavorable [−] SDH) for Stage I (**A**), Stage II (**B**), Stage III (**C**), and Stage IVa (**D**) esophageal cancer. Panels (**E**–**H**) depict overall survival among patients who did not achieve textbook oncological outcomes (TOO−), similarly stratified by SDH status for Stage I (**E**), Stage II (**F**), Stage III (**G**), and Stage IVa (**H**). Dashed line indicates median survival time.

Overall survival was 86% at 1 year and 43% at 5 years, with a median survival of 3.6 years. Patients with favorable SDHs (0–1 factors, denoted as SDH+) had a median survival of 4.0 years compared with 3.5 years for unfavorable SDHs (2–4 factors, denoted as SDH−). By stage-based survival analysis based on TOO status, across all stages, achieving TOO+ was associated with substantially improved survival, with median survival nearly doubling compared with TOO– patients. Unfavorable SDH status was consistently associated with worse survival among TOO– patients, particularly in stages II–III, while SDH effects were largely attenuated in patients achieving TOO+ (Table 3, Figure 2). Supplementary Table S1 reports the number of patients at risk at key follow-up intervals.

**Figure 3:**
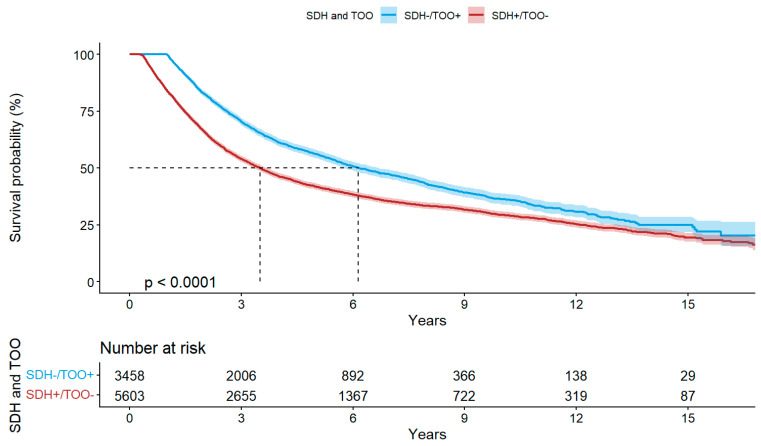
Comparison between social determinants of health (SDHs) and textbook oncologic outcomes (TOO) on overall survival. Footnote: Kaplan–Meier curves demonstrating overall survival stratified by combined SDH and TOO status. Patients were grouped by presentation with favorable SDHs (0–1 factors; SDH+) or unfavorable SDHs (2–4 factors; SDH–) and by achievement of textbook oncologic outcomes (TOO+ vs. TOO–). Survival differences illustrate the context of the relationship between SDHs and the quality of perioperative oncologic care, highlighting that patients with unfavorable SDHs who achieved TOOs had survival comparable to those with favorable SDHs, whereas failure to achieve TOOs was associated with worse survival regardless of SDH status.

To further illustrate the outcomes across contrasting SDH–TOO subgroups (Figure 3), patients with unfavorable SDHs who achieved TOOs (SDH–/TOO+) had survival comparable to those with favorable SDH, whereas patients with favorable SDHs who did not achieve TOOs (SDH+/TOO–) had markedly worse survival.

Before examining each SDH component in the multivariable model, we used uni- and multivariable analyses that include TOOs in the model (Supplementary Table S2), then stratified the model by TOO status (Table 4). In the TOO– cohort, higher SDH scores were significantly associated with worse survival with hazard ratios of 1.12 (95% CI, 1.06–1.19; *p* < 0.001) for score 2, 1.18 (95% CI, 1.10–1.26; *p* < 0.001) for score 3, and 1.55 (95% CI, 1.28–1.89; *p* < 0.001) for score 4, compared with score 0. In the TOO+ cohort, only SDH score 2 was associated with increased mortality (HR 1.15, 95% CI, 1.01–1.32; *p* = 0.038). Sensitivity analyses excluding stage IVa patients yielded similar results to the primary findings (Supplementary Tables S3 and S4).

**Figure 4:**
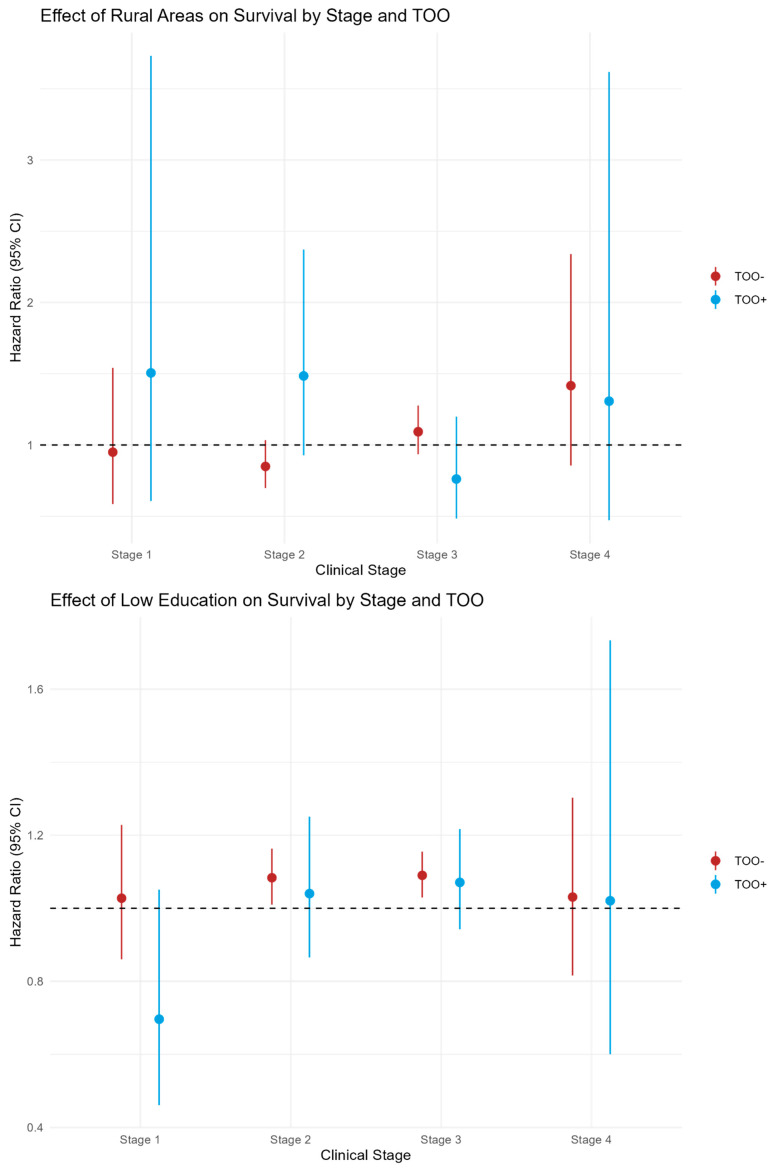

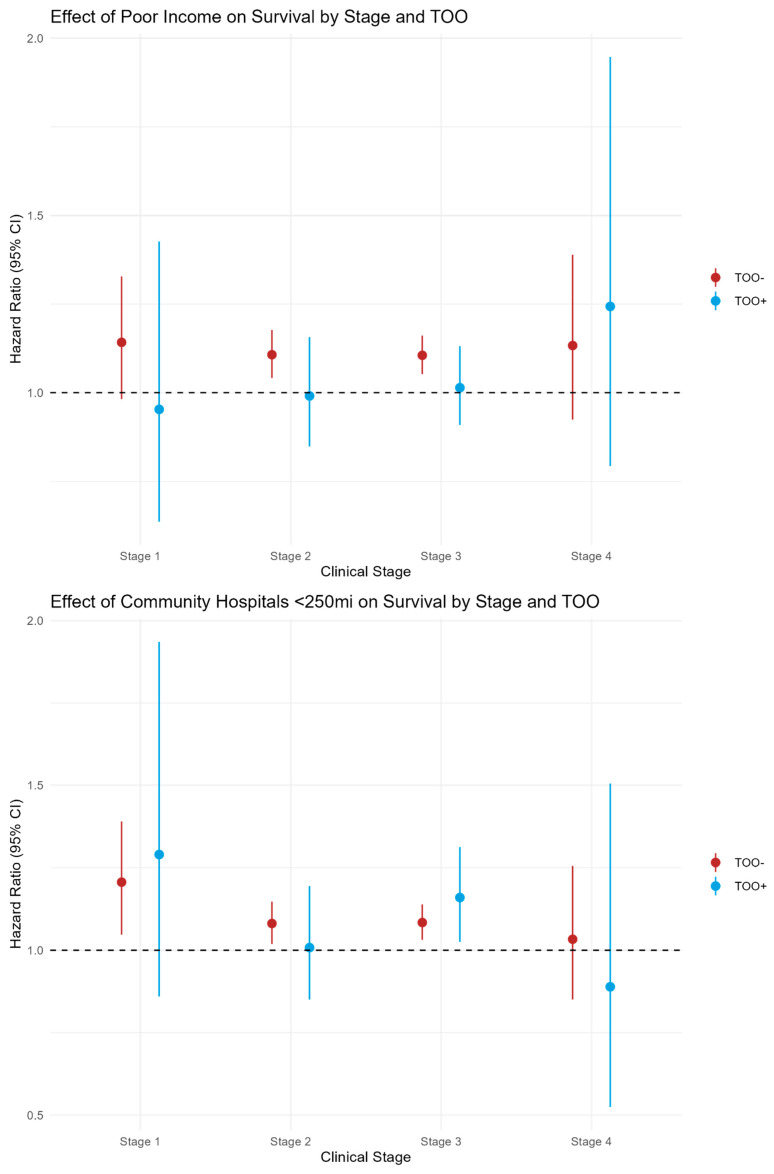
Forest plots showing adjusted HRs for SDHs by stage and TOOs (4 panels). 1 = null value, when there is no difference between the groups being compared.

Stage-stratified Cox regression analysis for each SDH component (Table 5, Figure 4) showed that in stage I, community hospital care (<250 miles) was associated with higher mortality in TOO– patients (HR 1.21, 95% CI: 1.05–1.39; *p* = 0.009), but no SDH factor reached significance in TOO+ patients. In stage II, community hospital care (<250 miles) was associated with higher mortality in TOO– patients (HR 1.08, 95% CI: 1.02–1.15; *p* = 0.010). In addition, poor income was significant in stage II TOO– patients (HR 1.10, 95% CI: 1.03–1.18; *p* = 0.008), while no SDH factors were significant in TOO+ patients. In stage III, community hospital care was associated with higher mortality in both TOO+ (HR 1.16, 95% CI: 1.03–1.31; *p* = 0.018) and TOO– (HR 1.09, 95% CI: 1.03–1.14; *p* = 0.001) patients, while poor income was additionally significant in TOO– patients (HR 1.09, 95% CI: 1.03–1.15; *p* = 0.003). In stage IVa, no SDH variable was statistically significant in either TOO group.

## 4. Discussion

In examining the interplay between TOOs and SDHs in shaping survival, several key findings emerged. First, only about 19% of patients achieved TOO+ status, and this subgroup demonstrated markedly improved survival compared with those who did not, confirming TOOs as a valid and clinically meaningful quality metric, which was previously established in other surgeries [19,20]. Second, while SDHs adversely influenced overall survival, as seen before [21,22,23], this effect was most pronounced among patients who failed to achieve TOOs. In contrast, patients who achieved TOO+ status appeared partially protected from the negative survival effects of poor income, low education, rural residence or travel to an optimal hospital. Third, the impact of SDHs varied by stage, with stages II and III showing the strongest associations, consistent with the treatment-sensitive biology of intermediate-stage EC. Finally, multivariable modeling demonstrated that among individual SDH factors, low income and care at community hospitals were the most consistent predictors of worse survival, even after accounting for comorbidities, insurance, and facility type. Together, these findings highlight the dual importance of achieving high-quality surgical and oncologic care, while also addressing structural inequities rooted in the social determinants of health.

### 4.1. Textbook Outcomes’ Effect on Survival in Esophageal Cancer

Our findings build on prior literature evaluating TOO in EC. The concept of TOO, originally defined as a composite outcome reflecting an ideal surgical pathway, has been validated in gastric and esophagogastric surgery, where it was shown to correlate with both perioperative safety and long-term survival [6,7,17]. Kalff et al. showed a 30% TOO achievement rate using older TOO criteria, but they still demonstrated that achieving TOO after esophagectomy was associated with superior survival across stages, even underscoring its prognostic value [6]. Our analysis confirms this relationship. Our analysis further showed that in several TOO– strata, particularly where survival curves are largely superimposable, the observed differences in median survival are likely driven by the very large sample size and resulting statistical power rather than representing a clinically meaningful effect. However, survival curve separation is clearly noticeable in our figures.

#### 4.1.1. Why Did Only 19% of Patients Achieve TOO+ Status?

TOOs are intentionally stringent, requiring optimal performance across multiple domains. Several factors contribute to the low achievement rate, including the fact that esophagectomy remains a complex, high-risk procedure with substantial morbidity [12]. Furthermore, the requirement for an extensive lymphadenectomy of at least 20 lymph nodes, based on recent surgical consensus, further limits achievement rates, as consistently attaining high lymph node yields after neoadjuvant therapy requires specialized surgical expertise and standardized pathology practices that are not uniformly available [14]. In addition, previous literature finds a meaningful proportion of patients do not receive timely adjuvant therapy, often due to complications or fragmented care [18], which can be reflected in the prolonged hospital stay in the TOO factors. Thus, the low nationwide rate of TOO+ underscores real-world variability in surgical quality and access to comprehensive care.

#### 4.1.2. Why Do Patients Who Achieve TOOs Have Better Survival than Those Who Do Not?

Patients who achieve TOOs are likely to experience smoother recovery, fewer delays, and greater access to multimodality care, factors that buffer against the barriers imposed by sociodemographic disadvantage as seen in previous studies. Merkow et al. demonstrated that postoperative complications reduce the likelihood of receiving adjuvant chemotherapy in pancreatic cancer [18], a principle that applies equally to EC. Thus, achieving TOO not only reflects high-quality surgery but also preserves treatment trajectories critical for long-term survival [7,24].

### 4.2. Social Determinants of Health’s Effect on Survival of Esophageal Cancer

The influence of SDHs in shaping cancer outcomes is well documented. Braveman and Gottlieb described SDHs as the “causes of the causes,” affecting both access to care and health outcomes [19]. In cancer care, lower income, lack of insurance, and rural residence are consistently associated with advanced stage at diagnosis, underutilization of guideline-based therapy, and inferior survival [2,20]. Halpern et al. demonstrated that uninsured and Medicaid patients presented at later stages across multiple cancer types, including EC [20]. Others showed that long-distance travel to high-volume centers improves survival after esophagectomy, highlighting the role of geographic access in determining outcomes [5,9,11]. Thus, patients who seek care at high-volume centers, even if the centers are located far from their place of residency, show improved survival.

#### 4.2.1. Why Did SDHs Affect Survival in TOO– but Not TOO+ Patients?

This pattern likely reflects several mechanisms based on TOO achievement. TOO+ patients complete the ideal care pathway. They receive adequate lymphadenectomy providing adequate staging, they experience fewer complications, and therefore they can receive timely and adequate adjuvant therapy, all of which buffer against socioeconomic and geographical barriers. Within the TOO+ cohort, only an SDH score of 2 reached statistical significance, while higher scores showed similar adverse trends (higher HRs) towards higher mortality but did not reach significance, likely due to limited sample size and event rates in the SDH 3–4 groups. On the other hand, TOO− patients are more vulnerable to unfavorable SDH-related barriers across all scores. While patients residing in low-SDH regions may travel to high-SDH centers for treatment, excluding such patients would reduce external validity, as cross-regional care seeking reflects real-world clinical practice. Furthermore, the composite score validates the results, including other factors such as education and income, which will remain constant regardless of where or whether the patients received surgical treatment. Other studies attempted to explain [2,19] the association of complications, readmissions, and incomplete treatment with resource limitations among disadvantaged patients, leading to delays or omission of adjuvant therapy, poorer follow-up adherence, or lower ability to travel for specialized care. This supports the idea that quality of care can ameliorate the burden of SDHs on survival, a phenomenon observed in other cancers [14,15,16].

#### 4.2.2. Difference Between the Composite SDH Score and Individual SDH Factors

Social barriers are increasingly recognized as shaping cancer outcomes. The first focus of SDH research was on individual components, such as income and travel distance to care centers, as key social factors of the clinical equation. However, a composite SDH score that captured cumulative disadvantage was later recognized as essential for evaluation of the impact of complex social factors, due to the synergistic impact of multiple socioeconomic and geographic factors [19]. Nonetheless, individual analysis of the SDH variables (income, hospital type, rurality, education) remains insightful to help uncover the specific mechanisms by which disparities arise. Our study shows that income and hospital type were the most powerful drivers of outcome disparities within TOO− patients. These two factors are probably the surrogates of the other SDH components, specifically for EC. Hospital access was defined as the presence of only community hospitals within 250 miles of a patient’s residence, reflecting limited access to tertiary or academic esophageal cancer centers. This measure was intended to capture restricted regional access to specialized, high-volume surgical care rather than travel distance itself. Patients traveling beyond this radius typically accessed higher-level referral centers, thereby mitigating access limitations. Patients from low-income areas may lack resources to travel to high-volume centers, afford supportive care, or manage treatment-related costs, which may not be related to level of education or rurality. Hospital type reflects structural differences in expertise, cancer pathways, and surgical volume [21]. Care at community hospitals may further compound these risks, as lower procedural volume and limited access to multidisciplinary expertise are well-established predictors of worse outcomes. A 2025 *JAMA Surgery* study revealed that patients who traveled more than 47 miles to high-volume centers for esophagectomy had improved 1-year and 5-year survival versus travelling < 7.6 miles for low-volume centers [11]. Thus, travel ability to well-qualified centers is crucial for EC, as has been shown among other thoracic cancers [22]. Different studies demonstrated that patients with higher education have an increased probability to receive curative treatment and adhere to the recommendations of the medical team [23,24]. When considering how individual factors explain poorer outcomes, we can hypothesize that education may affect comprehension of complex treatment plans as found in prior studies. Taken together, these factors show why patients with EC who live in socioeconomically disadvantaged or low-access areas experience inferior survival outcomes.

#### 4.2.3. SDH Impact on Survival Across Stage Strata

In EC stages II–III, where multimodality therapy (surgery plus chemotherapy or chemoradiation) is standard [25], sociogeographic barriers may exacerbate delays or underutilization of systemic therapy, amplifying survival disparities [2,26]. In contrast, stage I patients often do well with surgery alone, while stage IVa patients face poor prognosis regardless of social context, explaining why SDH effects were attenuated in these extremes. In our analysis, stage-stratified models revealed that SDH disadvantages substantially increased mortality among TOO− patients, particularly the individual factors of poor income and treatment at community hospitals, while the effect was largely attenuated among patients achieving TOO.

### 4.3. Other Factors Associated with Survival Across Stage/TOO Strata

Our results align closely with the literature and further extend it by demonstrating how the negative effects of SDHs are concentrated in TOO− patients, whereas TOO+ patients exhibit survival outcomes less dependent on sociodemographic background. In addition to the effects of SDH components, in our study, several clinical and treatment-related factors demonstrated important stage-specific associations with survival. Age was an independent adverse predictor in all TOO– cohorts and in stage II TOO+ patients, consistent with population-level data showing age-related decline in physiological reserve and treatment tolerance. Insurance status remained strongly associated with survival in TOO– patients across stages. Comorbidity burden (Charlson–Deyo score) was also a consistent and dose-dependent predictor of worse survival across nearly all stage/TOO strata, reflecting the impact of physical fitness and medical comorbidities on the overall outcomes after esophagectomy.

### 4.4. Implications and Actions Needed

The results of this study carry several actionable implications. First, it is critical to optimize surgical quality and to achieve TOOs. Ensuring that patients achieve TOO+ status should remain a quality metric for EC surgery. This applies across all hospitals, considering the low percentage of TOOs after EC surgery achieved by academic and high-volume centers. Collectively, our findings across thoracic malignancies suggest a consistent paradigm in which TOOs, a modifiable marker of care quality, can mitigate the adverse survival effects of SDHs, which are largely non-modifiable at the patient level. While SDHs exert a strong influence on survival when care pathways are suboptimal, its impact is attenuated among patients who achieve TOOs. This interaction highlights surgical quality and coordinated oncologic care as actionable levers to reduce socioeconomic disparities in thoracic cancer outcomes. Furthermore, quality improvement initiatives, adoption of enhanced recovery protocols, and centralization of care to high-volume academic centers may help increase TOO achievement. More recently, the use of robotic-assisted esophagectomy has been associated with improved TOO rates, with a propensity-matched analysis reporting double TOO+ achievement compared to other minimally invasive esophagectomies [4]. This could be in part due to improved lymphadenectomy, lower rate of complications and early discharge with low likelihood of readmission [27]. However, adoption of the robotic approach remains limited in low-volume centers, exacerbating existing inequities. In future studies, examining robotic esophagectomy in high-volume centers vs. low-volume centers would be warranted.

Second, SDH screening should be integrated into oncologic care. Identifying patients at risk due to poor income, limited education, or residence in underserved areas could allow for proper prognostication and targeted interventions [28]. Financial navigation, patient education, and enhanced communication strategies may mitigate some of these barriers [3].

Finally, it is possible to address geographic inequities in access to high-volume centers. Policies that incentivize referral to high-volume academic facilities, provide patient travel assistance, or expand telehealth and regional cancer networks could help reduce this disparity factor. Previous work has shown that centralization of surgical care improves survival not only in EC [11,21,22] but also in other malignancies such as pancreatic neuroendocrine tumors [16].

### 4.5. Strengths and Limitations

The strengths of this study include the large sample size from a national dataset, which enhances generalization, and the novel stratification of SDH effects by TOO status, providing insight into how surgical quality and social context are intertwined. The composite SDH score allowed for quantification of cumulative disadvantage. However, several limitations must be acknowledged. First, the retrospective design and reliance on the NCDB limit causal inference and are subject to reporting bias. Second, SDH variables were derived from ZIP code-level data rather than patient-level sociodemographic measures, which may underestimate heterogeneity within geographic areas. Third, information on novel therapies, cancer recurrence, and treatment adherence was not fully captured, limiting insight into post-surgical trajectories. Fourth, referral patterns and case-mix differences across regions are not captured and may influence TOO achievement and outcomes. Finally, while we stratified analyses by stage and TOO, residual confounding by unmeasured factors such as frailty, nutrition, and psychosocial support cannot be ignored.

## 5. Conclusions

In summary, this national analysis demonstrates that achievement of TOO after esophagectomy is strongly associated with improved survival and mitigates the effect of social determinants of health. Sociodemographic disadvantage exerted its greatest negative impact among patients who failed to achieve TOOs, particularly in stage II–III disease, whereas TOO+ patients at these stages experienced survival outcomes less dependent on SDHs. Low income and hospital type are the SDHs with the strongest impact in survival among patients that did not achieve TOOs. These findings highlight the dual necessity of optimizing surgical quality and addressing socioeconomic and sociodemographic inequities to improve outcomes in EC. Future interventions should prioritize centralization of care, targeted SDH mitigation strategies, and incorporation of TOOs into value-based cancer care frameworks.

## Figures and Tables

**Table 1:** Demographic and clinical characteristics stratified by TOO (row-wise percentages).

Characteristic	Overall *N* = 26,367 ^1^	TOO− *N* = 21,346 ^1^	TOO+ *N* = 5021 ^1^	*p*-Value ^2^
Age at Diagnosis	63 (9)	63 (9)	62 (9)	**<0.001**
Age (Groups)				**0.001**
<60	2169 (8.2%)	1727 (80%)	442 (20%)	
50–65	12,135 (46%)	9736 (80%)	2399 (20%)	
65–79	11,545 (44%)	9469 (82%)	2076 (18%)	
80+	518 (2.0%)	414 (80%)	104 (20%)	
Sex				0.10
Female	4265 (16%)	3491 (82%)	774 (18%)	
Male	22,102 (84%)	17,855 (81%)	4247 (19%)	
Race/Ethnicity				**0.002**
White	23,957 (91%)	19,349 (81%)	4608 (19%)	
Hispanic	775 (2.9%)	628 (81%)	147 (19%)	
Black	1049 (4.0%)	900 (86%)	149 (14%)	
Asian	390 (1.5%)	310 (79%)	80 (21%)	
Other	196 (0.7%)	159 (81%)	37 (19%)	
Insurance				**<0.001**
Private Insurance	11,962 (45%)	9495 (79%)	2467 (21%)	
Medicaid/Not Insured	1995 (7.6%)	1653 (83%)	342 (17%)	
Medicare/Other Government	12,410 (47%)	10,198 (82%)	2212 (18%)	
Charlson/Deyo Score				**0.039**
0	18,977 (72%)	15,282 (81%)	3695 (19%)	
1	5384 (20%)	4414 (82%)	970 (18%)	
2	1340 (5.1%)	1098 (82%)	242 (18%)	
3	666 (2.5%)	552 (83%)	114 (17%)	
Histology				**<0.001**
Adenocarcinoma	22,078 (84%)	17,720 (80%)	4358 (20%)	
Squamous CC	4289 (16%)	3626 (85%)	663 (15%)	
Clinical Stage				**<0.001**
Stage I	1649 (6.3%)	1390 (84%)	259 (16%)	
Stage II	9050 (34%)	7540 (83%)	1510 (17%)	
Stage III	14,505 (55%)	11,554 (80%)	2951 (20%)	
Stage IVa	1163 (4.4%)	862 (74%)	301 (26%)	
Primary Tumor Site				**<0.001**
Proximal esophagus	309 (1.2%)	268 (87%)	41 (13%)	
Mid esophagus	2318 (8.8%)	1927 (83%)	391 (17%)	
Distal esophagus	21,054 (80%)	16,913 (80%)	4141 (20%)	
Other	2686 (10%)	2238 (83%)	448 (17%)	
Facility Type				**<0.001**
Academic	13,080 (50%)	10,146 (78%)	2934 (22%)	
Integrated	4805 (18%)	4045 (84%)	760 (16%)	
Community	8482 (32%)	7155 (84%)	1327 (16%)	
Hospital Volume (High)				**<0.001**
High	16,888 (64%)	13,260 (79%)	3628 (21%)	
Low	9479 (36%)	8086 (85%)	1393 (15%)	

^1^ Mean (SD); *N* (%); ^2^ Wilcoxon rank sum test; Pearson’s chi-squared test; TOO: textbook oncological outcome. Bold indicates significance *p* < 0.05.

**Table 2:** SDH frequencies by clinical stage and TOO (column-wise percentage).

	TOO+, *N* = 5021				TOO−, *N* = 21,346			
Characteristic	Stage I *N* = 259	Stage II *N* = 1510	Stage III *N* = 2951	Stage IVa *N* = 301	Stage I *N* = 1390	Stage II *N* = 7540	Stage III *N* = 11,554	Stage IVa *N* = 862
Poor Income	167 (64%)	986 (65%)	1843 (62%)	199 (66%)	980 (71%)	5213 (69%)	7976 (69%)	617 (72%)
Community Hospitals < 250 mi	68 (26%)	357 (24%)	654 (22%)	59 (20%)	473 (34%)	2364 (31%)	3246 (28%)	270 (31%)
Low Education	195 (75%)	1196 (79%)	2285 (77%)	245 (81%)	1137 (82%)	6030 (80%)	9289 (80%)	705 (82%)
Rural Areas	10 (3.9%)	29 (1.9%)	59 (2.0%)	11 (3.7%)	28 (2.0%)	163 (2.2%)	245 (2.1%)	25 (2.9%)

*N* (%), TOO: textbook oncological outcome.

**Table 3:** Stage-specific survival by TOO and SDH status.

Characteristic	1 Year	5 Year	Median Survival
Overall	86% (85%, 86%)	43% (42%, 44%)	3.6 (3.6, 3.8)
SDH Score			
SDH+ (0–1)	87% (87%, 88%)	45% (44%, 47%)	4.0 (3.8, 4.2)
SDH− (2–4)	85% (84%, 85%)	42% (41%, 43%)	3.5 (3.4, 3.6)
Textbook Outcomes			
TOO−	82% (82%, 83%)	40% (39%, 41%)	3.2 (3.1, 3.3)
TOO+	100% (100%, 100%)	57% (55%, 58%)	6.4 (6.0, 6.8)
Clinical Stage			
Stage I	88% (87%, 90%)	53% (50%, 55%)	5.6 (5.0, 6.1)
Stage II	87% (86%, 87%)	46% (45%, 48%)	4.4 (4.2, 4.5)
Stage III	85% (84%, 85%)	40% (39%, 41%)	3.2 (3.1, 3.3)
Stage IVa	84% (81%, 86%)	39% (35%, 43%)	3.1 (2.8, 3.5)
Stage SDH/TOO			
Stage I/SDH−/TOO+	100% (100%, 100%)	69% (62%, 76%)	8.0 (6.9, 14)
Stage I/SDH+/TOO+	100% (100%, 100%)	66% (56%, 78%)	7.6 (6.0, —)
Stage I/SDH−/TOO−	85% (83%, 87%)	49% (45%, 52%)	4.8 (4.3, 5.4)
Stage I/SDH+/TOO−	89% (86%, 92%)	54% (48%, 59%)	6.0 (4.5, 7.4)
Stage II/SDH−/TOO+	100% (100%, 100%)	59% (56%, 63%)	7.3 (6.2, 8.1)
Stage II/SDH+/TOO+	100% (100%, 100%)	60% (56%, 66%)	7.5 (6.5, 10)
Stage II/SDH−/TOO−	84% (83%, 84%)	43% (42%, 45%)	3.8 (3.6, 4.1)
Stage II/SDH+/TOO−	86% (84%, 87%)	45% (43%, 48%)	4.1 (3.8, 4.5)
Stage III/SDH−/TOO+	100% (100%, 100%)	53% (51%, 56%)	5.6 (5.2, 6.1)
Stage III/SDH+/TOO+	100% (100%, 100%)	56% (53%, 60%)	6.5 (5.5, 7.9)
Stage III/SDH−/TOO−	80% (80%, 81%)	35% (34%, 36%)	2.8 (2.7, 2.9)
Stage III/SDH+/TOO−	82% (81%, 84%)	39% (37%, 41%)	3.0 (2.8, 3.2)
Stage IVa/SDH−/TOO+	100% (100%, 100%)	53% (44%, 63%)	5.1 (3.7, —)
Stage IVa/SDH+/TOO+	100% (100%, 100%)	56% (43%, 73%)	— (4.1, —)
Stage IVa/SDH−/TOO−	76% (73%, 80%)	33% (28%, 38%)	2.6 (2.3, 2.9)
Stage IVa/SDH+/TOO−	83% (78%, 88%)	37% (29%, 47%)	2.5 (2.2, 3.9)

SDH− denotes unfavorable social determinants of health (while SDH+ is favorable); TOO: textbook oncological outcome (achieved +, not achieved −).

**Table 4:** Multivariable factors associated with survival stratified by TOO status.

	Multivariable Analysis (TOO+)			Multivariable Analysis (TOO−)		
Characteristic	HR ^1^	95% CI ^1^	*p*-Value	HR ^1^	95% CI ^1^	*p*-Value
SDH Score						
0	—	—		—	—	
1	1.09	0.93, 1.27	0.3	1.06	0.99, 1.14	0.093
2	1.15	1.01, 1.32	**0.038**	1.12	1.06, 1.19	**<0.001**
3	1.10	0.92, 1.32	0.3	1.18	1.10, 1.26	**<0.001**
4	1.09	0.62, 1.92	0.8	1.55	1.28, 1.89	**<0.001**
Age at Diagnosis	1.01	1.00, 1.01	**0.001**	1.01	1.01, 1.02	**<0.001**
Sex						
Female	—	—		—	—	
Male	1.24	1.09, 1.41	**<0.001**	1.20	1.15, 1.26	**<0.001**
Private Insurance	0.90	0.82, 0.99	**0.030**	0.90	0.87, 0.94	**<0.001**
Clinical Stage						
Stage I	—	—		—	—	
Stage II	1.22	1.00, 1.48	0.051	1.15	1.07, 1.23	**<0.001**
Stage III	1.46	1.21, 1.77	**<0.001**	1.40	1.31, 1.51	**<0.001**
Stage IVa	1.44	1.10, 1.89	**0.009**	1.55	1.38, 1.74	**<0.001**
Adenocarcinoma (Ref: Squamous CC)	1.23	1.07, 1.41	**0.004**	1.02	0.97, 1.07	0.5
Distal Esophagus (Ref: Other Locations)	0.85	0.75, 0.95	**0.004**	0.87	0.84, 0.91	**<0.001**
Academic/Research Program	0.94	0.85, 1.04	0.2	0.97	0.93, 1.01	0.12
Hospital Volume						
High	—	—		—	—	
Low	1.07	0.96, 1.19	0.2	1.08	1.03, 1.12	**<0.001**

^1^ HR = hazard ratio; CI = confidence interval; TOO: textbook oncological outcome (achieved +, not achieved −). Bold indicates significance *p* < 0.05.

**Table 5:** Stage-stratified multivariable Cox models for individual SDH components stratified by TOO.

		TOO+			TOO−		
Group	Characteristic	HR	95% CI	*p*-Value	HR	95% CI	*p*-Value
Stage I	Poor Income	1.10	0.70, 1.73	0.7	1.14	0.96, 1.34	0.14
	Low Education	0.64	0.41, 1.02	0.063	0.95	0.78, 1.16	0.6
	Rural Areas	1.43	0.57, 3.58	0.4	0.93	0.58, 1.51	0.8
	Community Hospitals < 250 mi	1.35	0.89, 2.03	0.2	1.21	1.05, 1.40	**0.008**
	Age at Diagnosis	1.00	0.98, 1.03	0.7	1.02	1.01, 1.03	**<0.001**
	Private Insurance	0.67	0.44, 1.03	0.067	0.86	0.73, 1.00	0.055
	Charlson/Deyo Score = 0	0.84	0.55, 1.27	0.4	0.79	0.68, 0.92	**0.002**
Stage II	Poor Income	0.95	0.80, 1.14	0.6	1.10	1.03, 1.18	**0.007**
	Low Education	1.07	0.87, 1.32	0.5	1.03	0.95, 1.12	0.5
	Rural Areas	1.56	0.97, 2.50	0.066	0.84	0.69, 1.02	0.076
	Community Hospitals < 250 mi	1.00	0.84, 1.18	>0.9	1.08	1.02, 1.15	**0.010**
	Age at Diagnosis	1.01	1.00, 1.02	**0.009**	1.01	1.01, 1.02	**<0.001**
	Private Insurance	0.89	0.75, 1.06	0.2	0.90	0.85, 0.97	**0.003**
	Charlson/Deyo Score = 0	0.82	0.70, 0.96	**0.017**	0.84	0.79, 0.90	**<0.001**
Stage III	Poor Income	1.01	0.89, 1.14	>0.9	1.09	1.03, 1.15	**0.004**
	Low Education	1.07	0.93, 1.24	0.3	1.06	0.99, 1.13	0.11
	Rural Areas	0.72	0.46, 1.13	0.2	1.08	0.92, 1.26	0.4
	Community Hospitals < 250 mi	1.17	1.03, 1.32	**0.013**	1.09	1.03, 1.14	**<0.001**
	Age at Diagnosis	1.01	1.00, 1.01	0.11	1.01	1.01, 1.01	**<0.001**
	Private Insurance	0.93	0.82, 1.06	0.3	0.92	0.87, 0.97	**0.002**
	Charlson/Deyo Score = 0	0.78	0.69, 0.87	**<0.001**	0.90	0.86, 0.95	**<0.001**
Stage IVa	Poor Income	1.52	0.90, 2.56	0.12	1.18	0.93, 1.50	0.2
	Low Education	0.84	0.45, 1.56	0.6	0.94	0.71, 1.23	0.6
	Rural Areas	1.32	0.47, 3.66	0.6	1.24	0.75, 2.06	0.4
	Community Hospitals < 250 mi	0.91	0.53, 1.55	0.7	1.05	0.86, 1.27	0.7
	Age at Diagnosis	1.01	0.98, 1.03	0.5	1.01	1.00, 1.02	0.2
	Private Insurance	0.98	0.59, 1.62	>0.9	0.87	0.71, 1.08	0.2
	Charlson/Deyo Score = 0	0.93	0.58, 1.48	0.7	0.94	0.77, 1.14	0.5

Abbreviations: CI = confidence interval; HR = hazard ratio. Bold indicates significance *p* < 0.05.

## Data Availability

The raw data supporting the findings of this study are available from the National Cancer Database (NCDB) and may be accessed by investigators at Commission on Cancer-accredited institutions through the American College of Surgeons. Due to data use agreements and privacy restrictions, the data are not publicly available. All analyses were conducted using reproducible statistical code, which can be requested from the corresponding author A.A.

## Supplementary Materials

The following supporting information can be downloaded at: https://www.mdpi.com/article/10.3390/cancers18081253/s1, Table S1: Number of patients at risk at key follow-up intervals (1 and 5 years) for each subgroup. Table S2: Uni- and Multivariable Factors Associated with Overall Survival. Table S3: Uni- and Multivariable Factors Associated with Overall Survival without Stage IVa subgroup. Table S4: Multivariable Factors Associated with Survival Stratified by TOO Status without Stage IVa subgroup.

## Abbreviations

The following abbreviations are used in this manuscript:

ACS	American College of Surgeons
CDCI	Charlson–Deyo Comorbidity Index
CI	Confidence Interval
CoC	Commission on Cancer
EC	Esophageal Cancer
HR	Hazard Ratio
KM	Kaplan–Meier
MIE	Minimally Invasive Esophagectomy
NCDB	National Cancer Database
RAMIE	Robotic-Assisted Minimally Invasive Esophagectomy
SDH	Social Determinants of Health
SDH+	Favorable Social Determinants of Health
SDH−	Unfavorable Social Determinants of Health
TOO	Textbook Oncological Outcome
TOO+	Achieved Textbook Oncological Outcome
TOO−	Did Not Achieve Textbook Oncological Outcome
