# Supplementary material for: Survival Impact of Textbook Oncological Outcomes and SDHs for Patients with Operable Esophageal Cancer
Source: Cancers (Basel). 2026 Apr 15;18(8):1253. doi: 10.3390/cancers18081253 (PMC13114857; doi:10.3390/cancers18081253)
Supplement: Supplementary file 1 [file cancers-18-01253-s001.zip › cancers-4153597-supplementary.pdf]

Table S1. Number of patients at risk at key follow-up intervals (1 and 5 years) for each subgroup.

strata	1-year	5-year
Overall	22,284	7,671
0-1	6,189	2,223
2-4	16,095	5,448
0	17,339	5,955
1	4,945	1,716
Stage I	1,435	705
Stage II	7,751	3,318
Stage III	12,151	3,580
Stage IVa	947	68
Stage I / SDH+/TOO+	76	42
Stage I / SDH+/TOO-	295	147
Stage I / SDH-/TOO+	181	94
Stage I / SDH-/TOO-	883	422
Stage II / SDH+/TOO+	429	188
Stage II / SDH+/TOO-	1,661	726
Stage II / SDH-/TOO+	1,056	477
Stage II / SDH-/TOO-	4,605	1,927
Stage III / SDH+/TOO+	949	313
Stage III / SDH+/TOO-	2,529	789
Stage III / SDH-/TOO+	1,958	577
Stage III / SDH-/TOO-	6,715	1,901
Stage IVa / SDH+/TOO+	85	6
Stage IVa / SDH+/TOO-	165	12
Stage IVa / SDH-/TOO+	211	19
Stage IVa / SDH-/TOO-	486	31

Table S2. Uni- and Multivariable Factors Associated with Overall Survival.

	Univariable Analysis			Multivariable Analysis		
**Characteristic**	**HR[1]**	**95% CI[1]**	**p-value**	**HR[1]**	**95% CI[1]**	**p-value**
Poor Income	1.12	1.08, 1.16	**<0.001**			
Low Education	1.09	1.05, 1.13	**<0.001**			
Community Hospitals <250mi	1.12	1.08, 1.15	**<0.001**			
Rural Areas	1.04	0.93, 1.15	0.5			
SDH Score						
0	—	—		—	—	
1	1.08	1.01, 1.15	**0.018**	1.06	1.00, 1.13	0.054
2	1.14	1.08, 1.20	**<0.001**	1.13	1.07, 1.19	<0.001
3	1.26	1.19, 1.34	**<0.001**	1.17	1.10, 1.25	<0.001
4	1.59	1.32, 1.90	**<0.001**	1.49	1.24, 1.80	<0.001
Textbook Outcomes	0.60	0.57, 0.63	**<0.001**	0.60	0.58, 0.63	<0.001
Age at Diagnosis	1.02	1.01, 1.02	**<0.001**	1.01	1.01, 1.01	<0.001
Sex						
Female	—	—		—	—	
Male	1.19	1.14, 1.25	**<0.001**	1.21	1.16, 1.27	<0.001
Private Insurance	0.79	0.77, 0.82	**<0.001**	0.90	0.87, 0.93	<0.001
Clinical Stage						
Stage I	—	—		—	—	
Stage II	1.14	1.06, 1.22	**<0.001**	1.16	1.08, 1.24	<0.001

	Univariable Analysis			Multivariable Analysis		
Characteristic	HR[1]	95% CI[1]	p-value	HR[1]	95% CI[1]	p-value
Stage III	1.35	1.26, 1.44	**<0.001**	1.41	1.32, 1.51	<0.001
Stage IVa	1.42	1.28, 1.57	**<0.001**	1.52	1.37, 1.69	<0.001
Adenocarcinoma (Ref: Squamous CC)	1.03	0.99, 1.08	0.11	1.04	0.99, 1.09	0.10
Tumor Location (Distal esophagus)	0.91	0.87, 0.94	**<0.001**	0.87	0.84, 0.91	<0.001
Academic/Research Program	0.89	0.86, 0.92	**<0.001**	0.96	0.93, 1.00	0.055
Hospital Volume (High)						
High	—	—		—	—	
Low	1.14	1.10, 1.17	**<0.001**	1.07	1.03, 1.11	<0.001

[1]HR = Hazard Ratio, CI = Confidence Interval

Table S3. Uni- and Multivariable Factors Associated with Overall Survival without Stage IVa subgroup.

	Univariable Analysis			Multivariable Analysis		
Characteristic	HR	95% CI	p-value	HR	95% CI	p-value
Poor Income	1.12	1.08, 1.16	**<0.001**			
Low Education	1.09	1.05, 1.13	**<0.001**			
Community Hospitals <250mi	1.12	1.08, 1.15	**<0.001**			
Rural Areas	1.02	0.92, 1.14	0.7			
SDH Score						
0	—	—		—	—	
1	1.08	1.02, 1.15	**0.012**	1.07	1.00, 1.14	0.038
2	1.14	1.08, 1.21	**<0.001**	1.13	1.07, 1.19	<0.001
3	1.26	1.18, 1.34	**<0.001**	1.17	1.09, 1.25	<0.001
4	1.57	1.30, 1.89	**<0.001**	1.49	1.23, 1.80	<0.001
Textbook Outcomes	0.61	0.58, 0.63	**<0.001**	0.61	0.58, 0.64	<0.001
Age at Diagnosis	1.02	1.01, 1.02	**<0.001**	1.01	1.01, 1.02	<0.001
Sex						
Female	—	—		—	—	
Male	1.19	1.14, 1.24	**<0.001**	1.21	1.16, 1.27	<0.001
Private Insurance	0.79	0.77, 0.82	**<0.001**	0.90	0.87, 0.94	<0.001
Clinical Stage						
Stage I	—	—		—	—	
Stage II	1.14	1.06, 1.22	**<0.001**	1.16	1.08, 1.24	<0.001
Stage III	1.35	1.26, 1.44	**<0.001**	1.41	1.32, 1.51	<0.001
Adenocarcinoma (Ref: Squamous CC)	1.03	0.99, 1.07	0.2	1.04	0.99, 1.09	0.13
Tumor Location (Distal esophagus)	0.91	0.88, 0.94	**<0.001**	0.87	0.84, 0.91	<0.001
Academic/Research Program	0.89	0.86, 0.92	**<0.001**	0.96	0.92, 1.00	0.041
Hospital Volume (High)						
High	—	—		—	—	
Low	1.14	1.11, 1.18	**<0.001**	1.08	1.04, 1.12	<0.001

Abbreviations: CI = Confidence Interval, HR = Hazard Ratio

Table S4. Multivariable Factors Associated with Survival Stratified by TOO Status without Stage IVa subgroup.

	Multivariable Analysis (TOO+)			Multivariable Analysis (TOO-)		
**Characteristic**	**HR**	**95% CI**	**p-value**	**HR**	**95% CI**	**p-value**
SDH Score						
0	—	—		—	—	
1	1.08	0.92, 1.27	0.3	1.07	1.00, 1.15	0.059
2	1.14	0.99, 1.31	0.062	1.13	1.06, 1.20	<0.001
3	1.09	0.91, 1.31	0.3	1.17	1.09, 1.26	<0.001
4	1.06	0.59, 1.91	0.8	1.55	1.27, 1.90	<0.001
Age at Diagnosis	1.01	1.00, 1.01	0.002	1.01	1.01, 1.02	<0.001
Sex						
Female	—	—		—	—	
Male	1.22	1.07, 1.39	0.002	1.21	1.15, 1.27	<0.001
Private Insurance	0.90	0.81, 0.99	0.031	0.90	0.87, 0.94	<0.001
Clinical Stage						
Stage I	—	—		—	—	
Stage II	1.22	1.00, 1.49	0.051	1.15	1.07, 1.23	<0.001
Stage III	1.46	1.21, 1.77	<0.001	1.40	1.31, 1.51	<0.001
Adenocarcinoma (Ref: Squamous CC)	1.23	1.07, 1.41	0.004	1.01	0.96, 1.07	0.6
Tumor Location (Distal esophagus)	0.85	0.75, 0.95	0.005	0.88	0.84, 0.92	<0.001
Academic/Research Program	0.95	0.85, 1.05	0.3	0.96	0.92, 1.00	0.072
Hospital Volume (High)						
High	—	—		—	—	
Low	1.08	0.97, 1.20	0.2	1.08	1.04, 1.12	<0.001

Abbreviations: CI = Confidence Interval, HR = Hazard Ratio
